# Work-related risk factors for ulnar nerve entrapment in the Northern Finland Birth Cohort of 1966

**DOI:** 10.1038/s41598-021-89577-7

**Published:** 2021-05-11

**Authors:** Laura Miettinen, Jorma Ryhänen, Rahman Shiri, Jaro Karppinen, Jouko Miettunen, Juha Auvinen, Sina Hulkkonen

**Affiliations:** 1 Department of Hand Surgery, Helsinki University Hospital and University of Helsinki, Helsinki, Finland; 2 Finnish Institute of Occupational Health, Helsinki, Finland; 3 Medical Research Center Oulu, Oulu University Hospital and University of Oulu, Oulu, Finland; 4 Finnish Institute of Occupational Health, Oulu, Finland; 5 Center for Life Course Health Research, University of Oulu, Oulu, Finland

**Keywords:** Epidemiology, Risk factors

## Abstract

Ulnar nerve entrapment (UNE) is the second most common entrapment neuropathy in the upper extremity. The aetiology of UNE is multifactorial and is still not fully understood. The aim of the study was to identify occupational risk factors for UNE and to determine whether smoking modifies the effects of work-related factors on UNE. The study population consisted of the Northern Finland Birth Cohort of 1966 (NFBC1966). In total, 6325 individuals active in working life participated at baseline in 1997. Occupational risk factors were evaluated by a questionnaire at baseline. The data on hospitalizations due to UNE were obtained from the Care Register for Health Care between 1997 and 2018. The incidence rate of hospitalization due to UNE was 47.6 cases per 100,000 person-years. After adjusting for confounders, entrepreneurs (Hazard ratio (HR) = 3.68, 95% CI 1.20–11.27), smokers (HR = 2.51, 95% CI 1.43–4.41), workers exposed to temperature changes (HR = 1.72, 95% CI 1.00–2.93), workers with physically demanding jobs (HR = 3.02, 95% CI 1.39–6.58), and workers exposed to hand vibration (HR = 1.94, 95% CI 1.00–3.77) were at an increased risk of hospitalization for UNE. Exposure to work requiring arm elevation increased the risk of hospitalization due to UNE among smokers (HR = 2.62, 95% CI 1.13–6.07), but not among non-smokers. Work-related exposure to vibration and temperature changes, and physically demanding work increase the risk of hospitalization for UNE. Smoking may potentiate the adverse effects of work-related factors on UNE.

## Introduction

Upper extremity musculoskeletal disorders are common among the working population. They cause absenteeism, presenteeism, disability, and high health care costs^1^. They are more frequent among manual workers, which might partially be explained by high exposure to physical workload factors^2^.

Ulnar nerve entrapment (UNE) is the second most common entrapment neuropathy in the upper extremity after carpal tunnel syndrome^3^. Based on previous epidemiological studies, the annual incidence rate of UNE per 100,000 person-years ranges between 25 and 36 among men and between 17 and 26 among women^4–6^. The incidence rate increases until the middle age, before declining again, with the age peak of incidence varying around the fifth to seventh decade^4–7^.

The ulnar nerve most commonly becomes compressed at the elbow, in a site called cubital tunnel, and less frequently in the wrist, in Guyon’s canal^8^. The aetiology of UNE is multifactorial and still not fully understood. UNE is generally more common in males than in females^9^. Smoking tobacco has also been shown to be an independent risk factor for UNE^10–12^. The effects of metabolic and systemic disorders, such as diabetes and obesity on UNE are unclear, and the etiologic relationship between UNE and occupation has been debated.

The aim of this study was to determine the associations between occupational factors and UNE in a 21-year follow-up of a large birth cohort and to explore whether smoking modifies the effects of work-related factors on UNE.

## Methods

### Study population

**Figure 1:**
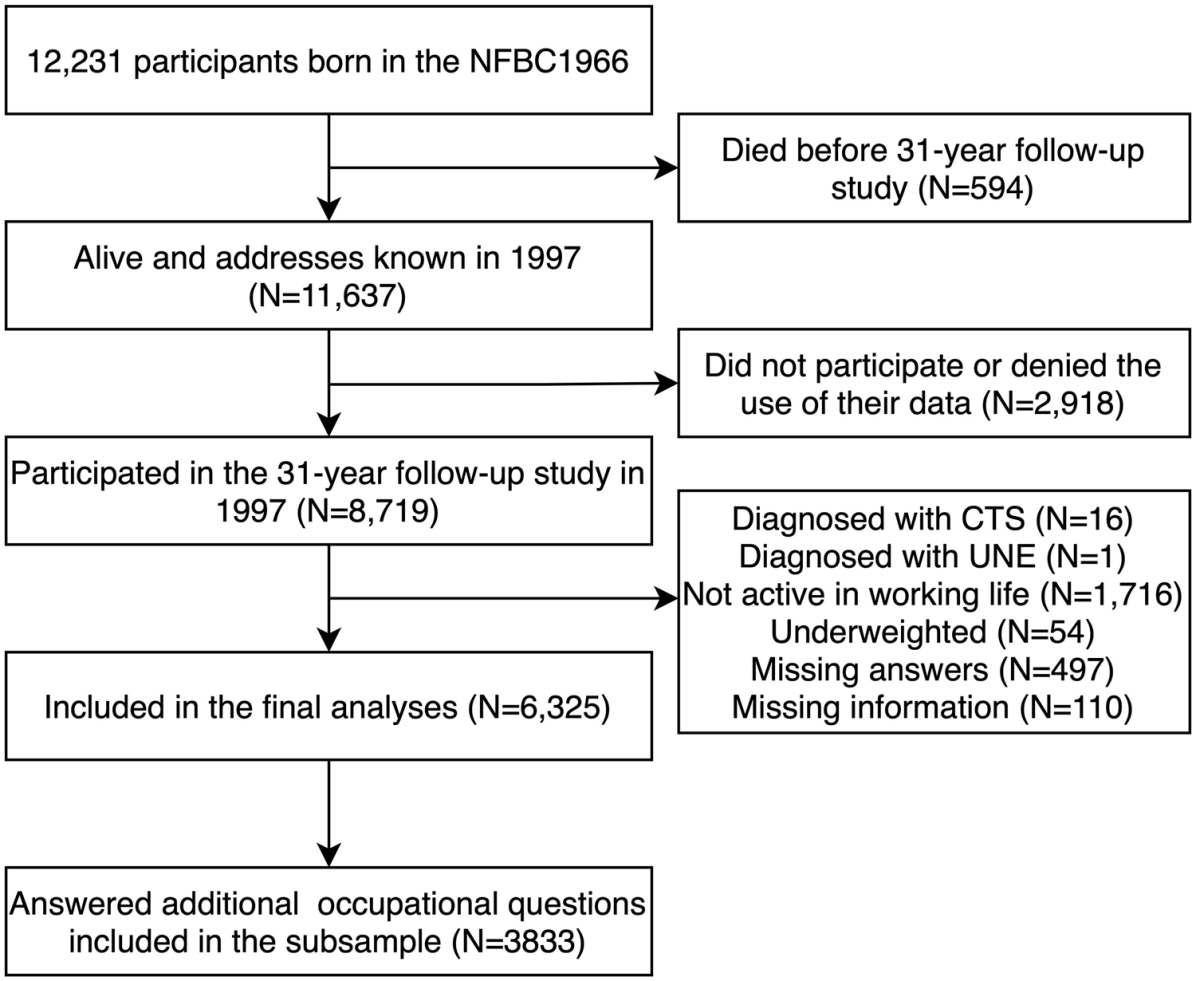
Flowchart of study population (the Northern Finland Birth Cohort of 1966, NFBC1966; carpal tunnel syndrome, CTS; ulnar nerve entrapment, UNE).

The study population consisted of the Northern Finland Birth Cohort of 1966 (NFBC1966); 12,231 individuals with an expected date of birth in 1966, born in the Oulu and Lapland provinces^13^. In 1997, the cohort population turned 31 years, and 8719 individuals participated in a follow-up study, giving their informed consent to voluntarily participation. Our analyses included NFBC1966 participants who had answered the postal questionnaire on work-related factors and were working ≥ 3 days a week in a paid job at study baseline in 1997. At baseline in 1997, one individual diagnosed with UNE and 16 diagnosed with carpal tunnel syndrome were excluded from the analyses. In addition, 607 individuals with missing data, 1716 individuals not being active in working life and 54 individuals who were underweight (BMI < 18.5 kg/m^2^ at the age of 31 years) were excluded. Of the final 6325 participants, 3833 answered an additional questionnaire on work-related factors during the clinical examination and formed a subsample (Fig. 1).

The participants’ personal identification numbers were replaced by study identification codes in the study data. The study was approved by the Ethics Committee of the Northern Ostrobothnia Hospital and followed the principles of the Declaration of Helsinki (as revised in 2008) of the World Medical Association.

### Hospitalization for ulnar nerve entrapment

As previously described by Hulkkonen et al.^14^, the study outcome was incident cases of diagnosed UNE that occurred after the baseline examination in 1997 until the end of 2018. The data on hospitalizations due to UNE were provided by the Care Register for Health Care, which is a national register that includes both public and private hospital data in Finland^15^. It contains information on patients’ demographics, diagnoses, surgical procedures, and dates of admission and discharge. The diagnoses are coded according to the International Classification of Diagnoses (ICD), and all ulnar entrapment neuropathies are coded under the same code. The diagnosis of UNE was coded 357.3 according to the eighth revision of ICD from 1969 to 1986, 354.2 according to the ninth revision of ICD from 1987 to 1995, and G56.2 according to the tenth revision of ICD from 1996 to 2018. The diagnoses were obtained from hospital data including both out- and inpatient services, with UNE as the primary or subsidiary diagnosis.

### Study variables

The data at baseline in 1997 were collected during a clinical examination and via a postal questionnaire. Occupational risk factors were evaluated by a postal questionnaire. All 6325 individuals answered the questions: ‘Are you exposed to the following in your work environment?’ with the different occupational exposures defined as heat, cold, temperature changes, and vibration in the hands. The study subsample of 3833 individuals also answered additional occupational questions: ‘Do you encounter the following in your work?’ with response options: heavy physical work, repetitive movements, lifting 1–15 kg objects, lifting > 15 kg objects, and working with arms elevated above shoulder level. The level of exposure was classified into none/light and moderate/heavy exposure.

Socio-economic status was defined on the basis of a questionnaire and according to Statistics Finland’s Classification of Socio-economic Groups 1989, which divides people’s socio-economic status into nine groups: farmers, entrepreneurs, upper-level employees, lower-level employees, manual workers, students, pensioners, unemployed, and unknown^16^. This classification takes into account the person’s stage in life, occupation and occupational activity. The categorical variable was formed by taking into account only those active in working life and classifying the variable into four categories: upper clerical workers, lower clerical workers, entrepreneurs, and farmers/manual workers. Socio-economic status coded as unknown was classified as missing data.

Body mass index (BMI, kg/m^2^), calculated from the individual’s height and weight measured during clinical examination or, if missing, from the information in the postal questionnaire, was classified as normal (BMI between 18.5 and 24.9 kg/m^2^) or overweight/obese (BMI ≥ 25 kg/m^2^). Fifty-four underweight individuals (BMI < 18.5 kg/m^2^) were excluded from the analysis. Based on smoking history, the study population was split into never-smokers and smokers, the latter included both current and past smokers. Information on diabetes, rheumatoid arthritis and thyroid diseases was collected from questionnaire at the baseline in 1997 (yes/no).

### Statistical analysis

The associations of background characteristics and occupational exposures with hospitalization for UNE were examined using the Cox proportional hazards regression model. All the variables that remained statistically significant in the univariable analyses were included in the multivariable Cox proportional hazards regression models. The analyses were conducted among the total sample and the subsample (N = 3833) with data on workload factors. Sex-specific univariable and multivariable analyses were also performed. First, we ran multivariable analyses for the variables that remained statistically significant in the univariable models. Second, non-significant variables were removed from the multivariable models one at the time until all variables were associated with UNE with a P value of ≤ 0.10. Kaplan–Meier estimator was used to display survival curves separately for male and female participants with and without physically demanding job. Log-rank test was used to test for difference in survival between two independent groups (with and without physically demanding job). Furthermore, a stratified analysis determined whether smoking modifies the associations between occupational physical exposures and hospitalization for UNE. Multiplicative interactions were also tested between occupational variables and smoking or BMI. We used the R program for statistical analysis.

## Results

Table 1 presents the baseline characteristics of the study population. At the age of 31, 23.4% of the participants were upper clerical workers, 35% were lower clerical workers, 7.6% were entrepreneurs and 33.9% were farmers or manual workers. Approximately 39% of the participants were overweight or obese, and 48.7% were past or current smokers. Over 1% of the participants had diabetes, 1.8% had thyroid disease and 0.8% had rheumatoid arthritis.

**Table 1:** Baseline characteristics of study population.

Characteristic	N	%
**Occupational class**		
Upper clerical workers	1483	23.4
Lower clerical workers	2213	35
Entrepreneurs	482	7.6
Farmers or manual workers	2147	33.9
**Body mass index**		
Normal	3886	61.4
Overweight/obese	2439	38.6
**Smoking**		
No	3244	51.3
Yes	3081	48.7
**Diabetes**		
No	6248	98.8
Yes	77	1.2
**Thyroid disease**		
No	6210	98.2
Yes	115	1.8
**Rheumatoid arthritis**		
No	6272	99.2
Yes	53	0.8
**Exposure to heat**		
None or light	5308	83.9
Moderate or high	1017	16.1
**Exposure to cold**		
None or light	5460	86.3
Moderate or high	865	13.7
**Exposure to temperature changes**		
None or light	4374	69.2
Moderate or high	1951	30.8
**Exposure to vibration to hands**		
None or light	5857	92.6
Moderate or high	468	7.4

The mean follow-up time was 21.3 ± 1.8 years. During the follow-up period, 64 participants were hospitalized for UNE (Table 2). Between 1997 and 2018, the annual incidence rate of hospitalization for UNE was 47.6 per 100,000 person-years.

**Table 2:** Univariable and multivariable hazard ratios (HR) with 95% confidence intervals (CI) of hospitalization for ulnar nerve entrapment in total study sample (N = 6325).

Characteristic	N	Cases	Univariable		Multivariable	
			HR	95% CI	HR*	95% CI
**Sex**						
Men	3259	35	1			
Women	3066	29	0.88	0.54–1.44		
**Occupational class**						
Upper clerical workers	1483	5	1		1	
Lower clerical workers	2213	20	2.71	1.02–7.22	2.17	0.81–5.83
Entrepreneurs	482	9	5.62	1.88–16.75	3.68	1.20–11.27
Farmers or manual workers	2147	30	4.20	1.63–10.83	2.23	0.82–6.06
**Body mass index**						
Normal	3886	36	1			
Overweight/obese	2439	28	1.24	0.76–2.04		
**Smoking**						
No	3244	17	1		1	
Yes	3081	47	2.97	1.71–5.17	2.51	1.43–4.41
**Diabetes**						
No	6248	64	NA			
Yes	77	0				
**Thyroid disease**						
No	6210	60	1		1	
Yes	115	4	3.57	1.30–9.82	4.14	1.50–11.44
**Rheumatoid arthritis**						
No	6272	64	NA			
Yes	53	0				
**Exposure to heat**						
None or light	5308	50	1			
Moderate or high	1017	14	1.47	0.81–2.66		
**Exposure to cold**						
None or light	5460	49	1			
Moderate or high	865	15	1.96	1.19–3.49		
**Exposure to temperature changes**						
None or light	4374	31	1		1	
Moderate or high	1951	33	2.40	1.47–3.92	1.72	1.00–2.93
**Exposure to vibration to hands**						
None or light	5857	51	1		1	
Moderate or high	468	13	3.13	1.70–5.76	1.94	1.00–3.77

*Adjusted for variables with P value ≤ 0.10.

**Figure 2:**
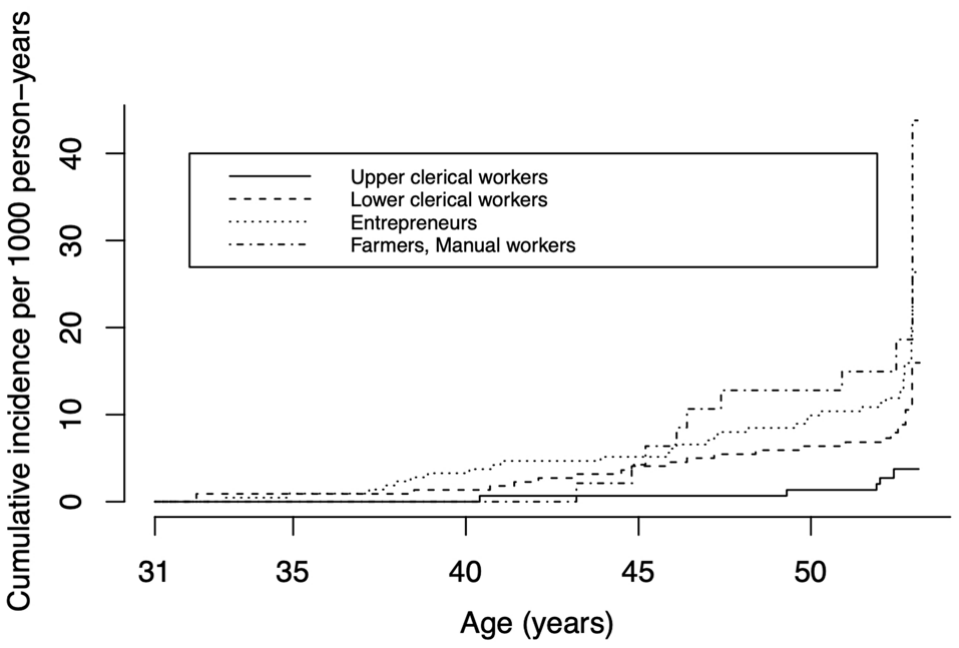
Cumulative incidence of hospitalization due to ulnar nerve entrapment (UNE) in different socio-economic groups over follow-up period. One individual was diagnosed with UNE before baseline in 1997.

Occupational class, smoking, thyroid disease, exposure to cold, and exposures to temperature changes and vibration to the hands were associated with UNE in the univariable analyses (Table 2, Fig. 2). The associations of gender, BMI and exposure to heat with UNE were not statistically significant. In the multivariable Cox’s proportional hazards regression model, occupational class, smoking, thyroid disease, exposure to temperature changes and exposure to vibration to the hands were statistically significantly associated with UNE (Table 2). In the sex-specific univariable analyses, occupational class, BMI ≥ 25 kg/m^2^, smoking, thyroid disease, exposures to cold and temperature changes and exposure to vibration to hands were associated with UNE among women and smoking, exposure to temperature changes and exposure to vibration to the hands were associated with UNE among men (Supplementary Table 1). In the sex-specific multivariable models, occupational class, BMI of ≥ 25 kg/m^2^, smoking, thyroid disease, and exposure to cold and vibration to the hands were associated with UNE among women; and smoking, exposure to temperature changes and exposure to vibration to the hands were associated with UNE among men (Supplementary Table 2).

**Figure 3:**
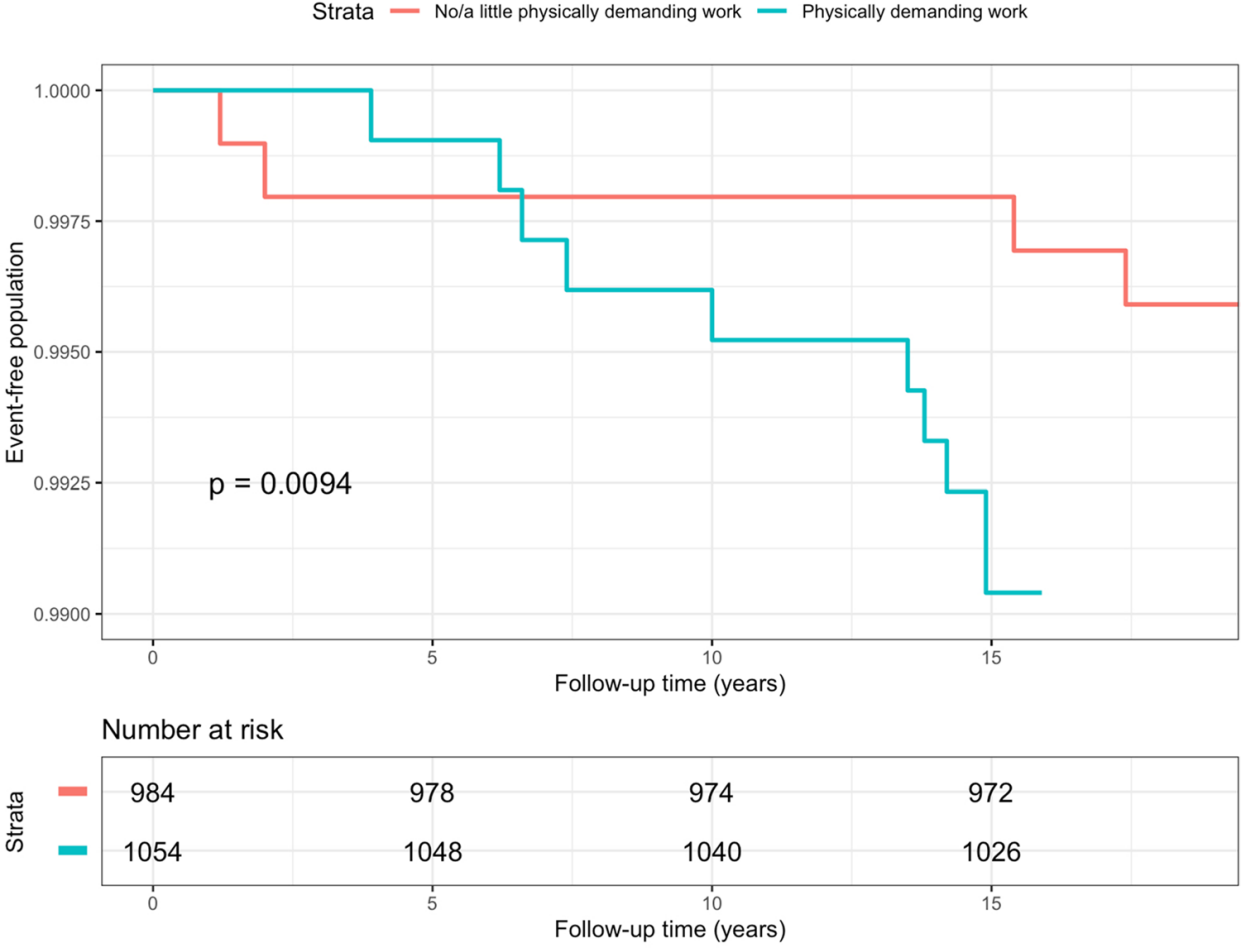
Kaplan–Meier curve of hospitalization for ulnar nerve entrapment among men in the subsample in follow-up 1997– 2018, stratified by physically demanding work at baseline. P-value for log-rank test.

**Figure 4:**
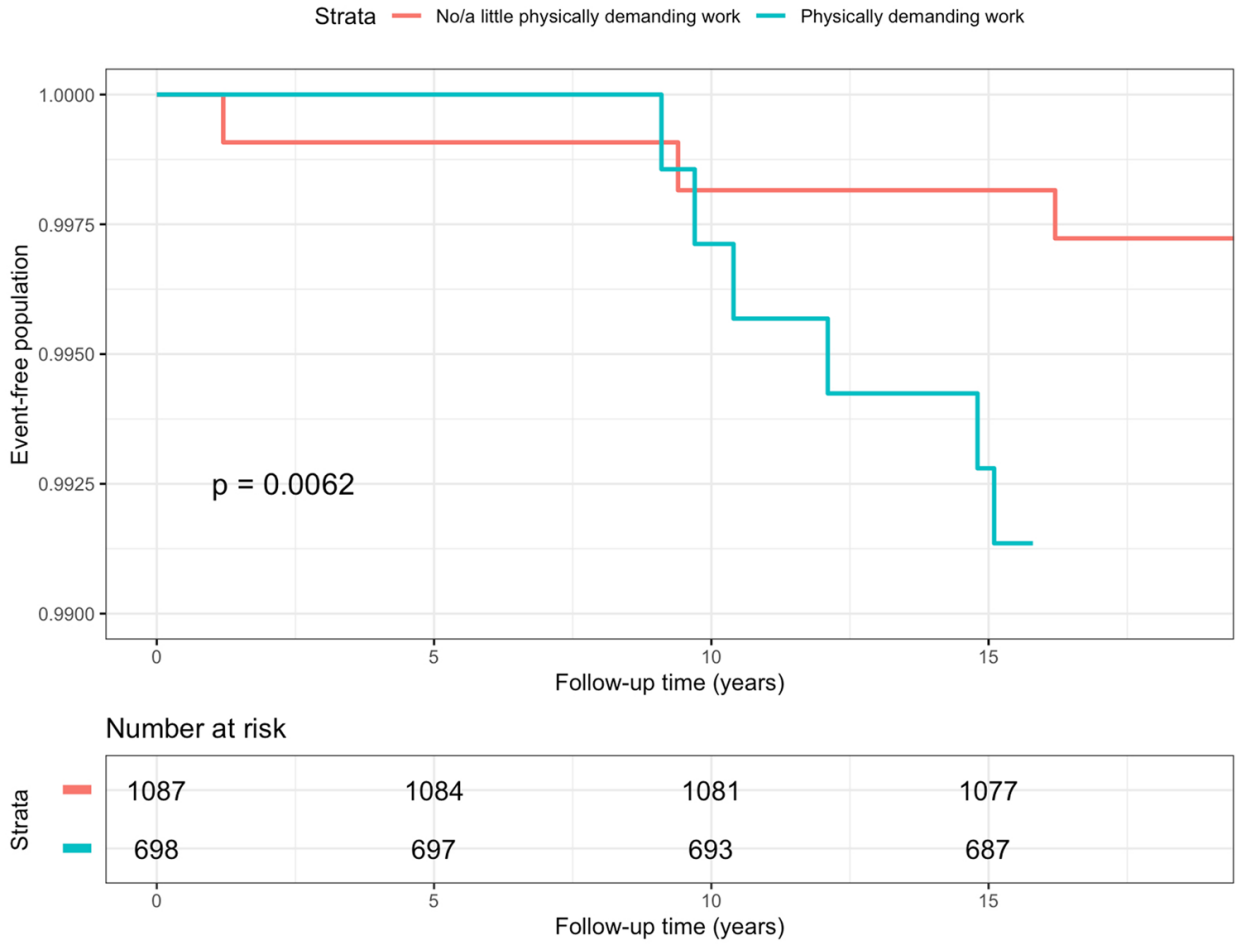
Kaplan–Meier curve of hospitalization for ulnar nerve entrapment among women in the subsample in follow-up 1997– 2018, stratified by physically demanding work at baseline. P-value for log-rank test.

Among the 3833 individuals in the subsample, physically demanding work, work requiring lifting and work requiring arm elevation at baseline were associated with the incidence of hospitalization for UNE in the univariable analyses (Table 3). Work demanding repetitive movements was not statistically significantly associated with hospitalization for UNE. In the multivariable model, physically demanding work and exposure to vibration to the hands at baseline were statistically significantly associated with hospitalization for UNE in the subsample (Table 3). Kaplan–Meier survival curves showed significant difference between participants with and without physically demanding job among both genders (Figs. 3 and 4). To assess the interaction between smoking and workload factors, a stratified analysis was conducted, and the study population was split into two groups on the basis of smoking status (Supplementary Tables 3, 4). Exposure to work requiring arm elevation increased the risk of hospitalization due to UNE among smokers (HR = 2.62, 95% CI 1.13–6.07), but not among non-smokers (Supplementary Table 4). There were no other statistically significant interactions between smoking or BMI and occupational risk factors.

**Table 3:** Univariable and multivariable hazard ratios (HR) with 95% confidence intervals (CI) of hospitalization for ulnar nerve entrapment in subsample (N = 3833).

Characteristic	N	Cases	Univariable		Multivariable	
			HR	95% CI	HR*	95% CI
**Physically demanding work**						
No	2073	9	1		1	
Yes	1760	29	3.84	1.82–8.12	3.02	1.39–6.58
**Lifting ≤ 15 kg**						
No	1583	9	1			
Yes	2250	29	1.27	1.08–4.80		
**Lifting > 15 kg**						
No	2374	15	1			
Yes	1459	23	2.52	1.31–4.83		
**Work requiring arm elevation**						
No	2666	16	1			
Yes	1167	22	3.19	1.67–6.07		
**Work demanding repetitive movements**						
No	836	5	1			
Yes	2997	33	1.85	0.72–4.74		
**Exposure to cold**						
None or light	3277	27				
Moderate or high	546	11				
**Exposure to temperature changes**						
None or light	2572	15				
Moderate or high	1811	23				
**Exposure to vibration to hands**						
None or light	3528	27			1	
Moderate or high	295	11			3.27	1.58–6.77

*Adjusted for variables with P value ≤ 0.10.

## Discussion

The present study found that using hand-held vibrating tools, physically demanding work and exposure to temperature changes increase the risk of hospitalization due to UNE. Furthermore, smoking potentiates the effect of work requiring arm elevation on UNE.

Only a limited number of epidemiologic studies have explored occupational risk factors for UNE. An epidemiological study conducted in Siena, Italy, reported a higher annual incidence of UNE among the residents of a subdistrict in which manual work was dominant^5^. A prospective cohort study with a 3-year follow-up found ‘holding a tool in a position’ to be the only predictor of UNE among workers whose occupations required repetitive work. Other exposures, such as ‘working with force’, ‘using a vibrating tool’ and ‘using elbows for support’ were non-significant. However, the study in question had only 15 incident cases of UNE, and the UNE diagnosis was based on clinical findings only^17^. In a case–control study, forceful work was associated with electrophysiologically confirmed UNE, with a potentially synergistic effect with non-neutral postures. The data on exposures and main job title were collected via a questionnaire, and a job exposure matrix was constructed to estimate job exposures. However, only 59% of the study participants responded to the questionnaire^18^. A prospective cohort study of male construction workers found that forceful work, static work, elbow leaning, and hand-arm vibration are associated with surgically treated UNE. However, no conclusion on vibration as a risk factor for UNE could be drawn on the basis of their data, as the usage of vibrating tools also required forceful hand-grip work^19^. Compared with the findings of these previous studies, our results from a large birth cohort reinforce the predisposing role of hand-arm vibration and physically demanding work in the development of UNE.

Several potential pathogenetic mechanisms have been considered to be behind biomechanical exposures and UNE. According to the literature, mechanical compression might induce intraneural oedema and functional changes that could result in impairment of nerve function^20^. The ulnar nerve is exposed to high levels of strain even with a normal range of upper limb motion^21^, and in an animal model, increase in strain caused reduction in the blood flow to the nerve and lead to ischemia^22^. When the elbow is flexed, the cross-sectional area of the cubital tunnel decreases and intraneural pressure increases^23–25^. The pressure inside the cubital tunnel has also been shown to increase during the contraction of the flexor carpi ulnaris muscle^26^. Vibration causes vasoconstriction, smooth muscle wall hypertrophy, periarterial fibrosis, and damage to the endothelial cells^27^. A histological model showed that tissue oedema and vasospasm from vibration leads demyelination and perineural fibrosis^28^. Exposure to vibration has also shown to increase the risk of carpal tunnel syndrome^29,30^.

In the current study, smokers exposed to work requiring arm elevation were at an increased risk for UNE. In line with the previous studies, this study shows that smoking increases the risk of UNE^10–12^. Smoking might decrease blood flow and induce changes in the myelin sheath leading to demyelination^31,32^. Smoking causes endothelial dysfunction and increases the production of free radicals, and may worsen the damage caused to peripheral nerves by ischemia^33^. Smoking posture and repetitive elbow flexion could increase strain and cause mechanical damage to the ulnar nerve. However, the preferred smoking hand does not correlated with the side of ulnar nerve entrapment^10,11^. When working with the arms elevated, the elbows are usually flexed to some degree, thereby increasing the strain and the pressure inside the cubital tunnel. In addition, intra-arterial blood pressure decreases with arm elevation^34^. In light of this, in the current study, work requiring arm elevation was only a risk factor among smokers. We speculated that the main mechanism in developing UNE might be circulatory.

Socio-economic status describes occupation and activity in working life. However, it does not describe specific biomechanical exposures, thus it might not be comparable with occupational exposures. In the current study population, the men worked more often as farmers or manual workers and the women as lower clerical workers. Compared with the participants with other socio-economic status, the entrepreneurs were at an increased risk of UNE. Workers with physically demanding jobs have higher demands for hand performance at work. They might seek help for their hand problems more often than individuals with non-physical jobs, as a less severe condition might reduce their ability to cope at work. The same may apply to entrepreneurs, as their livelihood may decline if their ability to work is reduced.

We found no previous studies that reported an association between temperature changes and ulnar nerve entrapment. Low temperature has shown to decrease nerve conduction velocity^35^. Our study population consisted of individuals born in northern Finland, where the climate and average temperature varies throughout the year.

Smoking and thyroid disease were associated with UNE in this study. However, the type of thyroid disease was not distinguished. An earlier study found hypothyroidism to be associated with UNE^36^. In the current study cohort, of 155 patients with thyroid disease at baseline, only four developed UNE during the follow-up period. Contrary to a previous study^9^, gender was not a risk factor for UNE.

To our knowledge, this is the first population-based study to examine the associations between occupational exposures and UNE. The longitudinal nature of the current study enabled us to assess the causal relationship. The NFBC1966 is a representative sample of a single-age birth cohort and represents the Finnish population with various socio-economic background well. The participation rate in the 31-year follow up study was very high, as 75% of the cohort participants had attended a clinical examination at baseline in 1997, and the follow-up time was long (1997–2018). We used the Care Register for Health Care which provides reliable data and recognizes over 80–99% of cases with common diagnoses^15^*.* We collected the Care Register for Health Care data from specialist care only. In addition, cases of UNE are electrophysiologically confirmed in Finland. Thus, false-positive diagnoses of UNE are less likely.

However, to our knowledge no previous study evaluated the validity of the Care Register for Health Care on hand surgical procedures in Finland, and recording of subsidiary diagnoses has been poor^15^. Unfortunately, all the diagnoses of ulnar entrapment neuropathies are coded under the same ICD code, so we cannot differentiate the level or handedness of UNE. Despite the large sample size of the cohort, a limitation of this study was its small number of incident cases and larger studies are needed to confirm these findings. The incidence of UNE peaks between the fifth and seventh decade, and in our study the follow-up time ended when the cohort population turned 52. In addition, we only used register data obtained from specialist care. This might explain the small number of cases in this cohort, as patients with only mild symptoms might have been treated in primary care. The data on occupational exposures was based on self-reported data and collected at the study baseline in 1997. Because of the long follow-up time, defining the causation between exposures at the baseline and the study outcome might be unreliable, as the exposures might have changed over time. Some study participants might have changed their occupation and exposed to different work-load factors during the follow-up period. This could have also caused crossover between socioeconomic groups. Furthermore, data on the daily exposure rates and the number of years exposed were not collected, which may have caused misclassification of the exposures. Overall, risk factors for UNE are still poorly recognized. Our study did not include all the potential risk factors for UNE, and we may have also missed some important confounding factors.

The aetiological relationship between UNE and occupation has been debated. Most published studies are case reports in specific occupations, and sufficient evidence that occupational exposure is a cause of UNE is lacking. Work disability among UNE patients is common and one population-based study revealed that half of UNE patients received wage replacements for over six months, and the mean medical and wage replacement cost of UNE averaged at almost 35,000 US dollars^37^. Further knowledge of occupational exposures is required for preventive measures. Early ergonomic interventions may reduce sick leaves^38^. Identifying occupational exposures and their connection with UNE is essential for patients to be able to receive occupational compensation.

In summary, physically demanding work, and exposures to vibration and temperature changes increase the risk of hospitalization for UNE. Smoking may potentiate the adverse effects of work-related factors on UNE. Further population-based studies are needed to confirm these findings.

## Supplementary Information

Supplementary Tables.

## Data Availability

The data that support the findings of this study are available from the Northern Finland Birth Cohorts, but restrictions apply to the availability of these data, which were used under license for the current study, and so are not publicly available. The authors have no right to share the data.
