# Supplementary material for: Work-related risk factors for ulnar nerve entrapment in the Northern Finland Birth Cohort of 1966
Source: Sci Rep. 2021 May 11;11:10010. doi: 10.1038/s41598-021-89577-7 (PMC8113547; doi:10.1038/s41598-021-89577-7)
Supplement: Supplementary file 1 — Supplementary Tables. [file 41598_2021_89577_MOESM1_ESM.pdf]

Work-related risk factors for ulnar nerve entrapment in the Northern Finland Birth Cohort of 1966

Laura Miettinen[1,*], Jorma Ryhänen[1], Rahman Shiri[2], Jaro Karppinen[3,4], Jouko Miettunen[3, 5], Juha Auvinen[3, 5], and Sina Hulkkonen[1]

Supplementary material

Characteristic	Men				Women			
	N	Cases	HR	95% CI	N	Cases	HR	95% CI
Occupational class								
Upper clerical workers	756	3	1		728	2	1	
Lower clerical workers	655	8	3.13	0.83-11.82	1561	12	2.83	0.63-12.65
Entrepreneurs	328	5	4.02	0.96-16.82	155	4	9.15	1.68-49.98
Farmers or manual workers	1528	19	3.23	0.96-10.92	627	11	6.41	1.42-28.92
Body mass index								
Normal	1711	22	1		2178	14	1	
Overweight/obese	1556	13	0.64	0.33-1.28	893	15	2.64	1.27-5.46
Smoking								
No	1471	9	1		1776	8	1	
Yes	1796	26	2.41	1.13-5.14	1295	21	3.77	1.67-8.53
Diabetes								
No	3238	35	-	-	3023	29	-	-
Yes	29	0			48	0		
Thyroid disease								
No	3246	35	-	-	2977	25	1	
Yes	21	0			94	4	4.99	1.73-14.34
Rheumatoid arthritis								
No	3251	35	-	-	3034	29	-	-
Yes	16	0			37	0		
Exposure to heat								
None or light	2650	27	1		2667	23	1	
Moderate or high	617	8	1.28	0.58-2.82	404	6	1.73	0.70-4.24
Exposure to cold								
None or light	2620	27	1		2847	22	1	
Moderate or high	647	8	1.22	0.56-2.69	224	7	4.10	1.75-9.61
Exposure to temperature changes								
None or light	1930	13	1		2446	18	1	
Moderate or high	1337	22	2.49	1.25-4.94	625	11	2.40	1.13-5.08
Exposure to vibration to hands								
None or light	2845	25	1		3032	26	1	
Moderate or high	422	10	2.65	1.27-5.52	51	3	6.90	2.09-22.8

Supplementary Table 1. Univariable sex-specific hazard ratios (HR) with 95% confidence intervals (CI) of hospitalization for ulnar nerve entrapment in total study sample (N= 6,338).

Characteristic	Men				Women			
	N	Cases	HR*	95% CI	N	Cases	HR*	95% CI
Occupational class								
Upper clerical workers					728	2	1	
Lower clerical workers					1561	12	2.22	0.49-9.97
Entrepreneurs					155	4	6.52	1.17-36.43
Farmers or manual workers					627	11	3.51	0.75-16.39
Body mass index								
Normal					2178	14	1	
Overweight/obese					893	15	2.06	0.98-4.35
Smoking								
No	1471	9	1		1776	8	1	
Yes	1796	26	2.18	1.02-4.67	1295	21	2.85	1.25-6.53
Thyroid disease								
No					2977	25	1	
Yes					94	4	3.90	1.32-11.50
Exposure to cold								
None or light					2847	22	1	
Moderate or high					224	7	2.83	1.143-7.03
Exposure to temperature changes								
None or light	1930	13	1					
Moderate or high	1337	22	2.01	0.98-4.14				
Exposure to vibration to hands								
None or light	2845	25	1		3032	26	1	
Moderate or high	422	10	1.91	0.88-4.12	51	3	3.03	0.86-10.71

*Adjusted for variables with P value ≤0.10

Supplementary Table 2. Sex-specific multivariable hazard ratios (HR) with 95% confidence intervals (CI) of hospitalization for ulnar nerve entrapment in total study sample (N=6,338).

Characteristic	Non-smokers (N=1955)				Smokers (N=1878)			
	N	Cases	HR	95% CI	N	Cases	HR	95% CI
Physically demanding work								
No	1132	3	1		941	6	1	
Yes	823	6	2.77	0.69-11.06	937	23	3.87	1.58-9.52
Lifting ≤15 kg								
No	901	2	1		682	7	1	
Yes	1054	7	3.00	0.62-14.46	1196	22	1.77	0.76-4.15
Lifting >15 kg								
No	1318	3	1		1056	12	1	
Yes	637	6	4.16	1.04-16.62	822	17	1.82	0.87-3.81
Work requiring arm elevation								
No	1419	6	1		1247	10	1	
Yes	536	3	1.31	0.33-5.26	631	19	3.82	1.78-8.22
Work demanding repetitive movements								
No	484	1	1		352	4	1	
Yes	1471	8	2.67	0.33-21.33	1526	25	1.43	0.50-4.10

Supplementary Table 3. Univariable hazard ratios (HR) with 95% confidence intervals (CI) of hospitalization for ulnar nerve entrapment in subsample (N=3,833), stratified by smoking.

Characteristic	Non-smokers (N=1955)				Smokers (N=1878)			
	N	Cases	HR*	95% CI	N	Cases	HR*	95% CI
Physically demanding work								
No					941	6	1	
Yes					937	23	2.49	0.93-6.67
Work requiring arm elevation								
No					1247	10	1	
Yes					631	19	2.62	1.13-6.07

*Adjusted for variables with P value ≤0.10

Supplemental Table 4. Multivariable hazard ratios (HR) with 95% confidence intervals (CI) of hospitalization for ulnar nerve entrapment in subsample (N=3,833), stratified by smoking.
